# Development of Magnesium and Siloxane-Containing Vaterite and Its Composite Materials for Bone Regeneration

**DOI:** 10.3389/fbioe.2015.00195

**Published:** 2015-12-02

**Authors:** Shinya Yamada, Akiko Obata, Hirotaka Maeda, Yoshio Ota, Toshihiro Kasuga

**Affiliations:** ^1^Department of Frontier Materials, Graduate School of Engineering, Nagoya Institute of Technology, Nagoya, Japan; ^2^Yabashi Industries Co., Ltd., Ogaki, Japan

**Keywords:** bioceramics, magnesium, calcium, silicate, bone regeneration

## Abstract

Development of novel biomaterials with Mg^2+^, Ca^2+^, and silicate ions releasability for bone regeneration is now in progress. Several inorganic ions have been reported to stimulate bone-forming cells. We featured Ca^2+^, silicate, and especially, Mg^2+^ ions as growth factors for osteoblasts. Various biomaterials, such as ceramic powders and organic–inorganic composites, that release the ions, have been developed and investigated for their cytocompatibilities in our previous work. Through the investigation, providing the three ions was found to be effective to activate osteogenic cells. Magnesium and siloxane-­containing vaterite was prepared by a carbonation process as an inorganic particle that can has the ability to simultaneously release Ca^2+^, silicate, and Mg^2+^ ions to biodegradable polymers. Poly (l-lactic acid) (PLLA)- and bioactive PLLA-based composites containing vaterite coatings were discussed regarding their degradability and cytocompatibility using a metallic Mg substrate as Mg^2+^ ion source. PLLA/SiV composite film, which has a releasability of silicate ions besides Ca^2+^ ion, was coated on a pure Mg substrate to be compared with the PLLA/V coating. The degradability and releasability of inorganic ions were morphologically and quantitatively monitored in a cell culture medium. The bonding strength between the coatings and Mg substrates was one of the key factors to control Mg^2+^ ion release from the substrates. The cell culture tests were conducted using mouse osteoblast-like cells (MC3T3-E1 cells); cellular morphology, proliferation, and differentiation on the materials were evaluated. The PLLA/V and PLLA/SiV coatings on Mg substrates were found to enhance the proliferation, especially the PLLA/SiV coating possessed a higher ability to induce the osteogenic differentiation of the cells.

## Introduction

Various types of bioactive ceramics and glasses have been investigated for application in bone regeneration (Jarcho, 1981; Winter et al., 1981; LeGeros, 2002). Calcium phosphate and calcium silicate glasses, such as Bioglass^®^ 45S5, are well known to have excellent bioactivity and promote new bone formation *in vivo*. Recently, several ions released from these materials have been found to influence cell functions and some of the ions can accelerate osteogenesis, angiogenesis, and antibacterial activity (Hoppe et al., 2011). Calcium (Ca^2+^) ions released from composite materials, consisting of a type II collagen gel and hydroxyapatite (HA), have been demonstrated to have a stimulatory effect on the activation of mouse primary osteoblasts (Maeno et al., 2005). Ca^2+^ ion concentrations of 2–4 mM are reported to be suitable for enhancing the proliferation and survival of osteoblasts, whereas concentrations of 6–8 mM favor their differentiation and biomineralization of extracellular matrix (ECM). Ca^2+^ ion concentrations >10 mM were found to be cytotoxic for cells.

Stimulatory effects for the enhancement of bone formation were found for the soluble silica species and Ca^2+^ ions that were released from Bioglass^®^ 45S5 (Xynos et al., 2000a). The cellular numbers of human osteoblasts (HOBs) cultured in the ionic products of Bioglass^®^ 45S5, obtained by its dissolution in Dulbecco’s modified eagle medium (DMEM), increased by 155.1 ± 6.5% compared with normal DMEM after 4 days of culture. HOBs cultured on the Bioglass^®^ 45S5 disk exhibited higher alkaline phosphatase (ALP) activity, which is known to be associated with osteoblastic differentiation of HOBs, compared with those grown on a bioinert (plastic) substrate, after 6 days of culture (Xynos et al., 2000b). Trace amounts of Ca^2+^ and silicate ions are believed to be beneficial for the promotion of bone formation.

Additionally, magnesium (Mg^2+^) ions have been reported to enhance cell adhesion to materials, along with the differentiation and biomineralization of osteoblasts. The expression of various integrin family members, which are a class of adhesion proteins, was increased on Mg^2+^-modified alumina compared with Mg^2+^-free controls (Zreiqat et al., 2002). The stimulatory effects of Mg^2+^ ions on early bone cell differentiation have also been reported, whereby osteoblasts cultured on bioactive SiO_2_–CaO–P_2_O_5_–MgO glass exhibited a high ALP activity (Saboori et al., 2009). Moreover, the effects of Mg^2+^ ions on angiogenic function have been clarified by Maier et al. (2004). Mg^2+^ ions stimulate the proliferation of human umbilical vein endothelial cells (HUVECs) and enhance the mitogenic response to angiogenic factors. These stimulatory effects of the released inorganic ions on cellular activities should be beneficial to the design of new biomaterials for bone regeneration.

Magnesium- and siloxane-containing vaterite (MgSiV) has been developed as a material that provides Mg^2+^, Ca^2+^, and silicate ions upon degradation in our previous work (Yamada et al., 2014a). Of the calcium carbonates, vaterite, calcite, and aragonite, vaterite is the most thermodynamically unstable polymorph. The chemical structure and their degradation behavior in physical condition were examined. From cell culture tests, mouse osteoblast-like cells had an improved proliferation, differentiation, and mineralization in the extract of the MgSiV and the dependence on the ion-type contained in the extract; these cell functions were significantly enhanced when all of the ions, Mg^2+^, Ca^2+^, and silicate ions, were simultaneously provided to the cells.

The improved functions of the cells were also observed in the results of the cell culture tests for metallic magnesium (Mg) substrates coated with a siloxane-containing vaterite (SiV) and poly (l-lactic acid) (PLLA) composite layer (Yamada et al., 2013, 2014b). The metallic Mg substrate coated with the composite layer releases the three kinds of ions at the same time; Ca^2+^ and silicate ions are supplied by SiV, and Mg^2+^ ions are from the metallic Mg substrates. The cell proliferation and differentiation were accelerated on the metallic Mg substrate coated with the composite layer in comparison with those on the sample releasing only Ca^2+^ and Mg^2+^ ions or no ions.

The up-regulation effects by the three kinds of ions on the cells were found to be similar even though the providing process was different between the MgSiV and the Mg substrate coated with the composite layer. These findings imply that biomaterials providing the three kinds of ions would be good for achievement of the rapid mineralization of osteogenic cells. In addition, such inorganic ions supplied by bioceramics can be regarded to be one of the important factors for promoting bone formation *in vivo*.

In this review, we provide an overview of materials providing Mg^2+^, Ca^2+^, and silicate ions, i.e., the MgSiV and the Mg substrates coated with PLLA/SiV composite layer, and osteoblast-like cell reactions to the materials. In addition, new composite materials that possess an excellent 3D structure (cotton wool-like structure), flexibility, and a providing ability of Ca^2+^ and silicate ions are introduced as well. They have been expected to be good candidates for bone fillers.

## Magnesium- and Siloxane-Containing Vaterite

The development of SiV (Nakamura et al., 2013) and its composites with biodegradable polymer, such as PLLA or poly(lactic-*co*-glycolic acid) (PLGA), as materials providing Ca^2+^ and silicate ions has been published in our previous work (Obata et al., 2009, 2010; Wakita et al., 2010; Fujikura et al., 2013). Electrospun fibremats consisting of the PLLA/SiV composites possessed excellent cell compatibility *in vitro* and a formation of mineralized tissue *in vivo*. Especially, in the results of cell culture tests, the PLLA/SiV composites accelerated the proliferation and the differentiation of mouse osteoblast-like cells in comparison with a composite consisting of vaterite and PLLA (Obata et al., 2009). This implies that the ions released from the PLLA/SiV composites, particularly silicate ions, must contribute to the enhanced cell functions. Many reports demonstrated that such ions are able to enhance osteogenic cell functions, proliferation, differentiation, and mineralization, and regarded to be one of the important factors for bone formation in the body (Hoppe et al., 2011). Thus, the SiV-containing composites are expected to be some of the good candidates for new biomaterials promoting bone formation.

In contrast, the ions released from the SiV and its composites are believed to have no up-regulation effect on cell adhesion. Cell adhesion is a significant process of progressing proliferation for adherent cells, such as osteoblasts and fibroblasts. To improve cell adhesion should be useful to improve proliferation and following biological reactions of these cells. Mg^2+^ ions have been found to improve cell adhesion to substrate surfaces (Zreiqat et al., 2002). Thus, to incorporate magnesium to the SiV was expected to achieve new biomaterials having higher cell compatibility, along with enhanced cell adhesion, proliferation, differentiation, and mineralization. In addition, MgSiV is expected to possess buffering action in aqueous solution since it releases carbonate ions as well, while most of silica-based bioactive glasses, such as 45S5-type bioactive glass, increase its surrounding pH. This might be good for cells cultured on the material surfaces. The preparation of MgSiV powders has been reported in our previous work (Yamada et al., 2014a). In the present short review, some of their significant results are introduced briefly.

### Preparation

Magnesium and siloxane-containing vaterite powders were synthesized by a carbonation process in methanol using calcium hydroxide, 3-aminopropyltriethoxysilane (APTES), and magnesium hydroxide as calcium, silicate, and magnesium sources, respectively (Yamada et al., 2014a). All the chemicals were mixed into the slurry under carbon dioxide gas flow, resulting in the formation of a precursor gel. The obtained gel was aged for 12 h at room temperature, dried at 110°C for 24 h, and then grounded to form particles. The obtained MgSiV contained 2.0 wt% of magnesium and 2.8 wt% of silicon. SiV powders were also prepared by the same method without adding magnesium source.

### Structure

**Figure 1:**
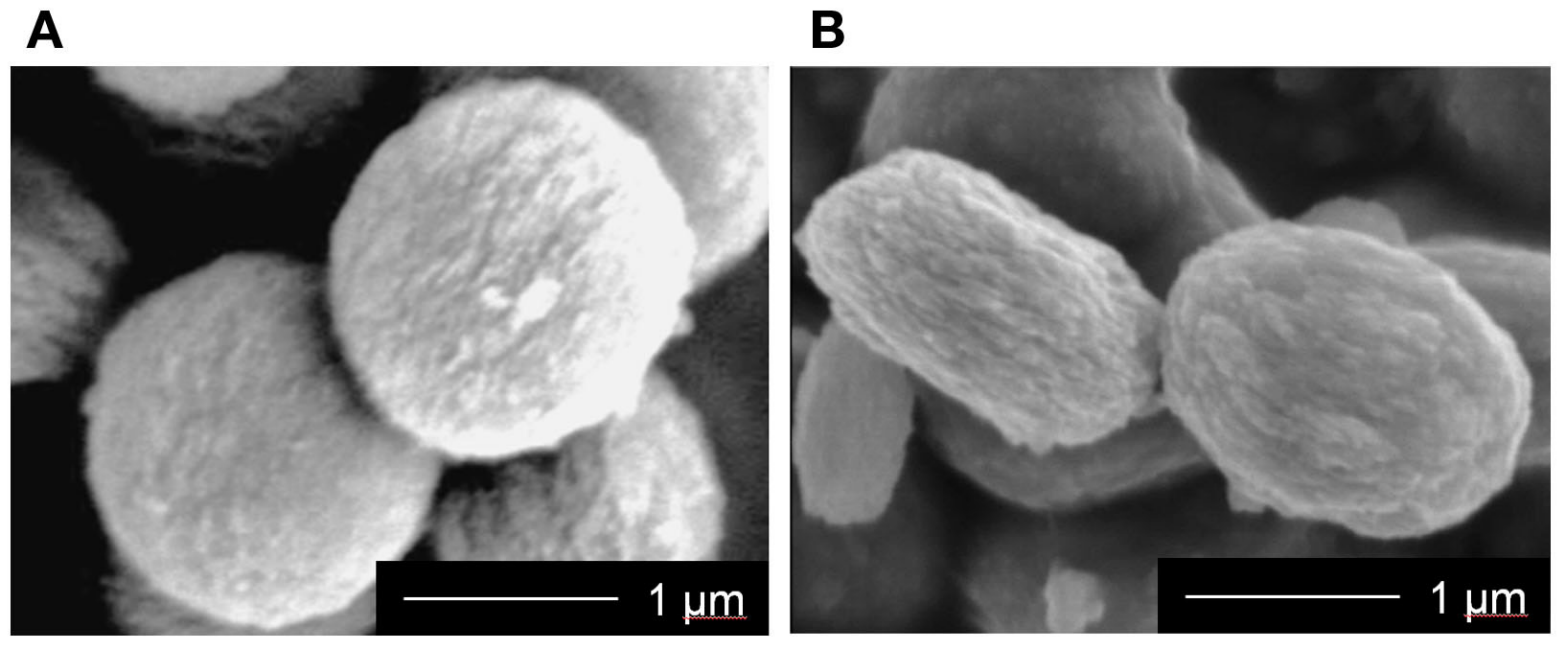
**SEM images of (A) SiV and (B) MgSiV**. Reprinted with permission from Yamada et al. (2014a).

The prepared MgSiV samples exhibit flat-spherical morphology, around 1.3 μm in diameter and 0.6 μm in thickness (Figure 1). They consist of primary particles with several being 10 nm in size. On the other hand, the SiV have a spherical morphology, ~1.4 μm in diameter. The reason why the morphologies are different between the two samples is that it is expected that the orientation of the vaterite phase in MgSiV might be varied by Mg^2+^ ions. Vaterite is known to have a characteristic symmetry and orientation of carbonate ions in its crystalline structure. The orientation is parallel to the *c*-axis (Wang and Becker, 2009). The siloxane derived from APTES is believed to contribute to the stabilization of the *c*-face (Nakamura et al., 2013). The Mg^2+^ ions in the MgSiV might influence the orientation, resulting in the formation of the flat-spherical particles. The surface areas of the two samples are also different; they were 103 and 34 m^2^/g for the MgSiV and SiV, respectively, from the results of BET–nitrogen adsorption.

**Figure 2:**
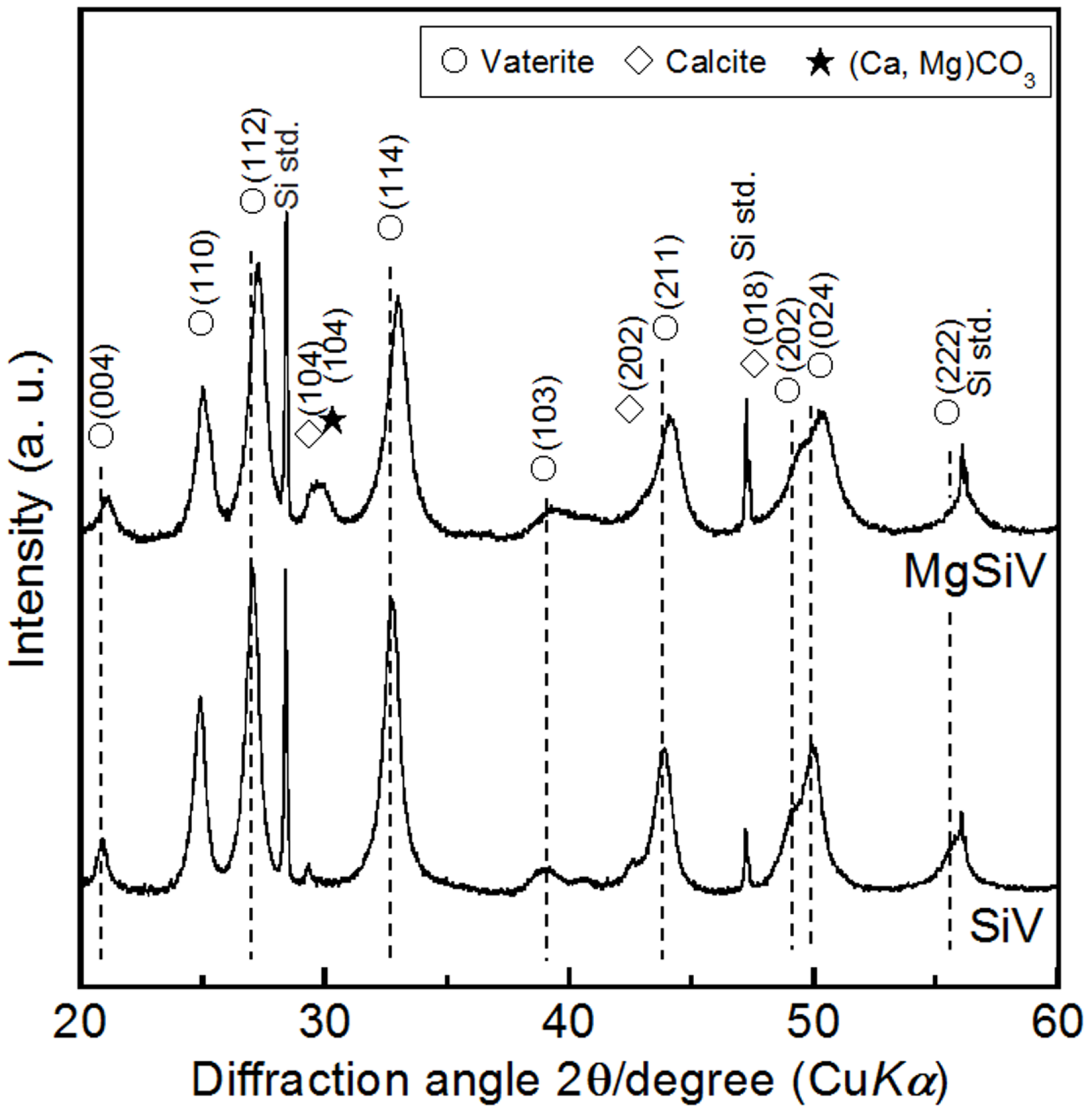
**XRD patterns of SiV and MgSiV**. Reprinted with permission from Yamada et al. (2014a).

The crystalline phase of MgSiV consists predominantly of vaterite one and contains small amounts of calcite and calcium magnesium carbonate. X-ray diffraction (XRD) pattern of the MgSiV demonstrates that the peaks corresponding to the *c*-axis-dependent plane of vaterite shifted to a higher angle (Figure 2). By contrast, the *ab* plane shows no shift. Mg might be incorporated into the vaterite crystalline structure and substitute for some of the Ca-sites in vaterite, since the lattice spacing for the vaterite (004) plane changed from 0.426 to 0.421 nm by adding Mg to SiV. The MgSiV and SiV were found to also contain the amorphous calcium carbonate (ACC) phase in their structures from the results of Fourier transform infrared spectroscopy (FTIR) analysis (data not shown here).

### Ion release

**Figure 3:**
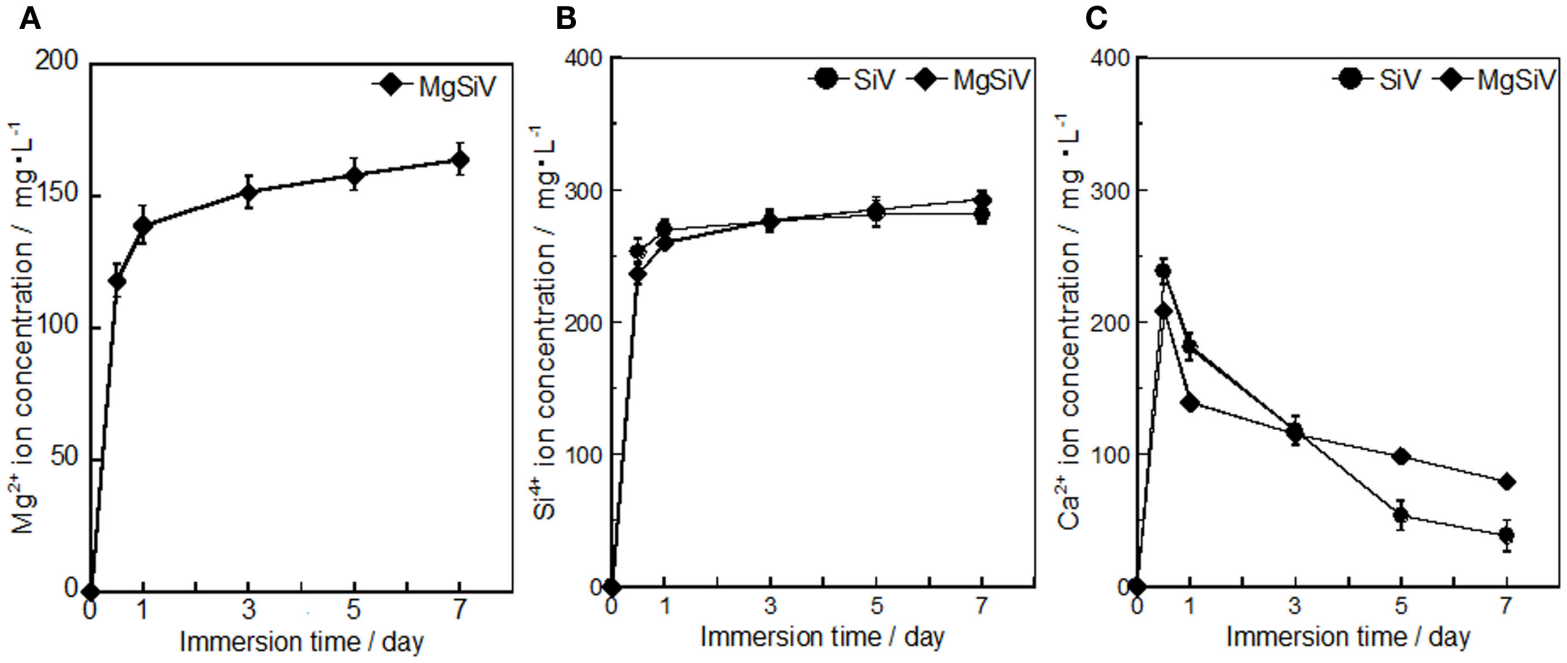
**Amounts of (A) Mg, (B) Si, and (C) Ca elements dissolved from SiV and MgSiV**. Reprinted with permission from Yamada et al. (2014a).

**Figure 4:**
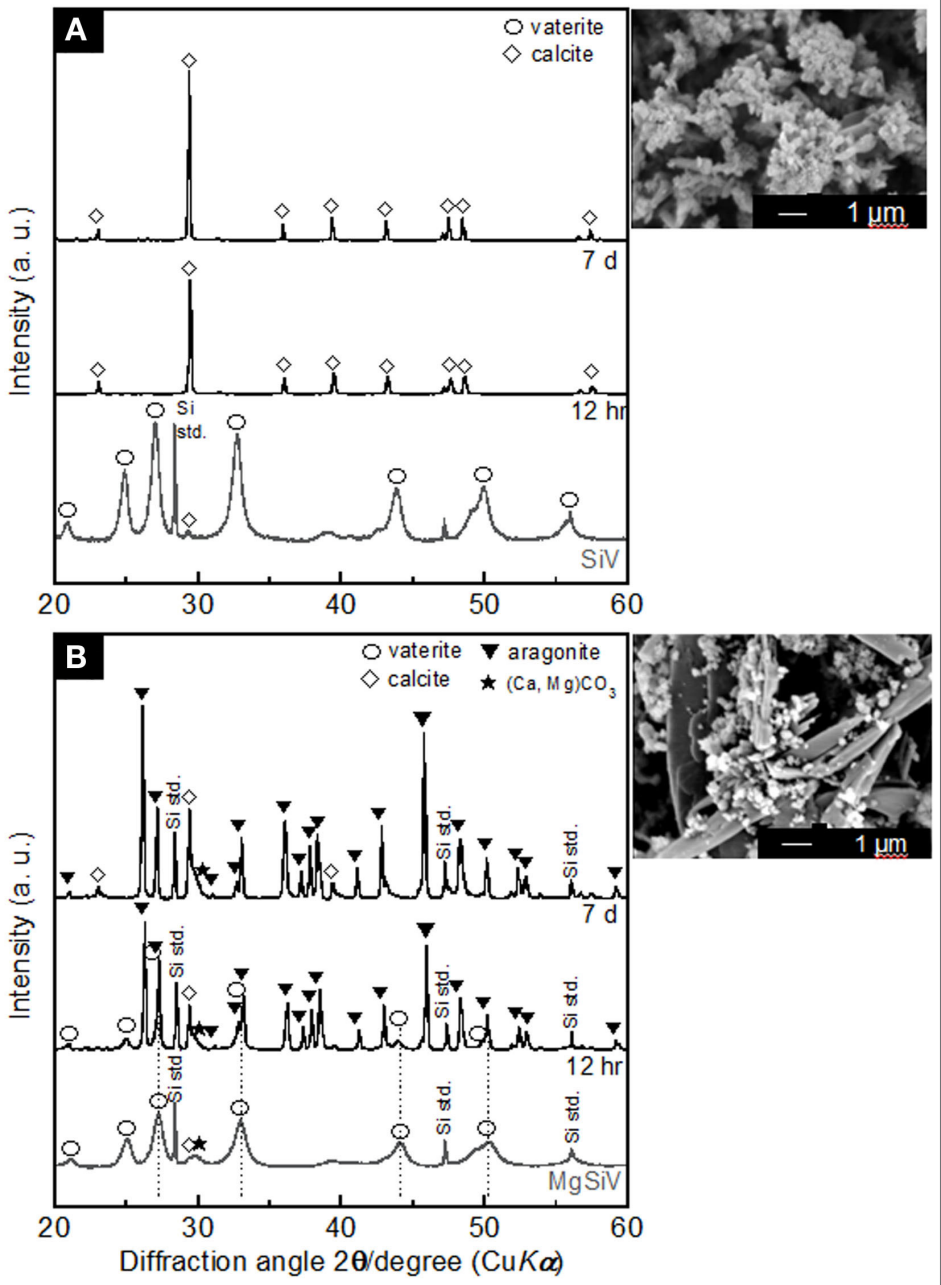
**XRD patterns of (A) SiV and (B) MgSiV before and after soaking in Tris buffer solution (pH 7.4) and their SEM images after 7 days of the soaking**. Reprinted with permission from Yamada et al. (2014a).

The MgSiV powders release Mg^2+^, Ca^2+^, and silicate ions through their crystalline transformation from vaterite to aragonite phase in aqueous solution. They were immersed in the Tris–HCl buffer solution (pH 7.4) for 7 days, and the amount of the released ions was measured by inductively coupled plasma atomic emission spectroscopy (ICP-AES) (Figure 3). Their crystalline phases at each time point during the immersion were characterized by XRD (Figure 4). The crystalline phase of the MgSiV transformed from vaterite into aragonite in 12 h after the immersion and simultaneously released 60% of the total Mg and 80% of the total Si. The release of the two ions continued until day 7, while the release rate decreased after 12 h. A total amount of 83% of the total Mg and almost all Si in the MgSiV were released in the 7 days. On the other hand, the Ca-release behavior was different from those of Mg and Si. The amount of the released Ca was maximum after 12 h and then continued to decline until day 7. The increase in the Ca amount in 12 h after the immersion is believed to originate from the dissolution of ACC. On the other hand, the decline in the amount is due to the formation of precipitates at the bottom of the containers used.

The SiV powders possess ion-release behavior similar to the MgSiV. The transformation of the crystal phase of the SiV is, however, different from the MgSiV; its phase changed from vaterite to calcite in 12 h after the immersion. This is because aragonite phase precipitates more easily in an aqueous solution containing a large amount of Mg^2+^ ions (Kitano, 1962; Bischoff, 1968; Sawada et al., 1990; Böttcher et al., 1997; Morse et al., 1997; Kitamura, 2001; Zhang et al., 2012). No Mg^2+^ ion is incorporated in the lattice of aragonite because it has a tightly bound hydration shell (Falini et al., 1996, 2009). After 12 h, small peaks corresponding to vaterite phase are still seen for the MgSiV, while the crystal phase of SiV completely transformed to calcite. Mg must be incorporated into the vaterite crystalline structure in the MgSiV, since the peaks corresponding to vaterite in the MgSiV shifted compared with those of the SiV. The Mg incorporated into the vaterite dissolved from the MgSiV in 12 h, because the peaks revert to the original positions of the SiV. Vaterite disappeared and the predominant crystalline phase was aragonite after 7 days. The particle shape of the MgSiV varied after the immersion; no original MgSiV particles were found, but needle-like ones, which is a typical shape of aragonite, were newly observed in the samples after 7 days of immersion.

## PLLA/SiV Composite Coating on a Metallic Magnesium Substrate

Metallic Mg and its alloys possess biodegradability and proper mechanical properties and are regarded to be good candidates for metallic biomaterials (Staiger et al., 2006; Witte et al., 2008; Witte, 2010). They have the suitable properties for being used as vascular stents or orthopedic implants; they possess high reactivity with water and dissolve in body fluid through corrosion, which would contribute to the avoidance of secondary surgery after healing and achieve a complete replacement of bone tissue. In addition, they have the similar Yong’s modulus (41–45 GPa) to that of human cortical bone, which might contribute to the decrease of bone resorption around the implants. The modulus is lower than that of any other metallic biomaterials, such as titanium alloys (Staiger et al., 2006).

On the other hand, there are concerns that metallic Mg rapidly degrades and produces corrosion, hydroxyl ions, and bubbles of hydrogen gas around the surrounding tissues (Witte et al., 2005). This induces an extremely high local alkali concentration (pH > 9.0) on the metallic Mg surface, which is harmful for cells (Shen et al., 2012). The bubbles of hydrogen gas formed in 1 week after implantation, which induced vacant spaces around the metallic Mg. This is attributed to poor integration of the metallic Mg implanted into body tissue (Witte et al., 2005). To solve these problems, the metallic Mg surfaces were coated with biodegradable polymer, such as poly(ε-caprolactone) (PCL) and PLLA (Wong et al., 2010; Xu and Yamamoto, 2012). The cytocompatibility of the metallic Mg was improved by the polymer coatings.

Bioactive coatings consisting of PLLA-based composites containing SiV or vaterite (V) powders have been developed in our previous work, since Mg^2+^, Ca^2+^, and silicate ions must be provided from the metallic Mg, vaterite phase, and siloxane in the SiV, respectively, which were expected to enhance osteogenic cell activities. The adhesion, proliferation, and differentiation of MC3T3-E1 cells cultured on the prepared samples were estimated to clarify the effects of the each ion released from the samples on the cell functions (Yamada et al., 2013, 2014b).

### Preparation

The SiV and V powders were prepared by a carbonation method aforementioned. The composites of PLLA and SiV or V were prepared by a melt-blending method, dissolved in chloroform, and then coated on surfaces of a commercially available pure metallic Mg with a spin coater. The amount of SiV or V in the composites was set to be 60 wt% (~47 vol%).

### Morphology, Bonding Strength, and Degradation

**Figure 5:**
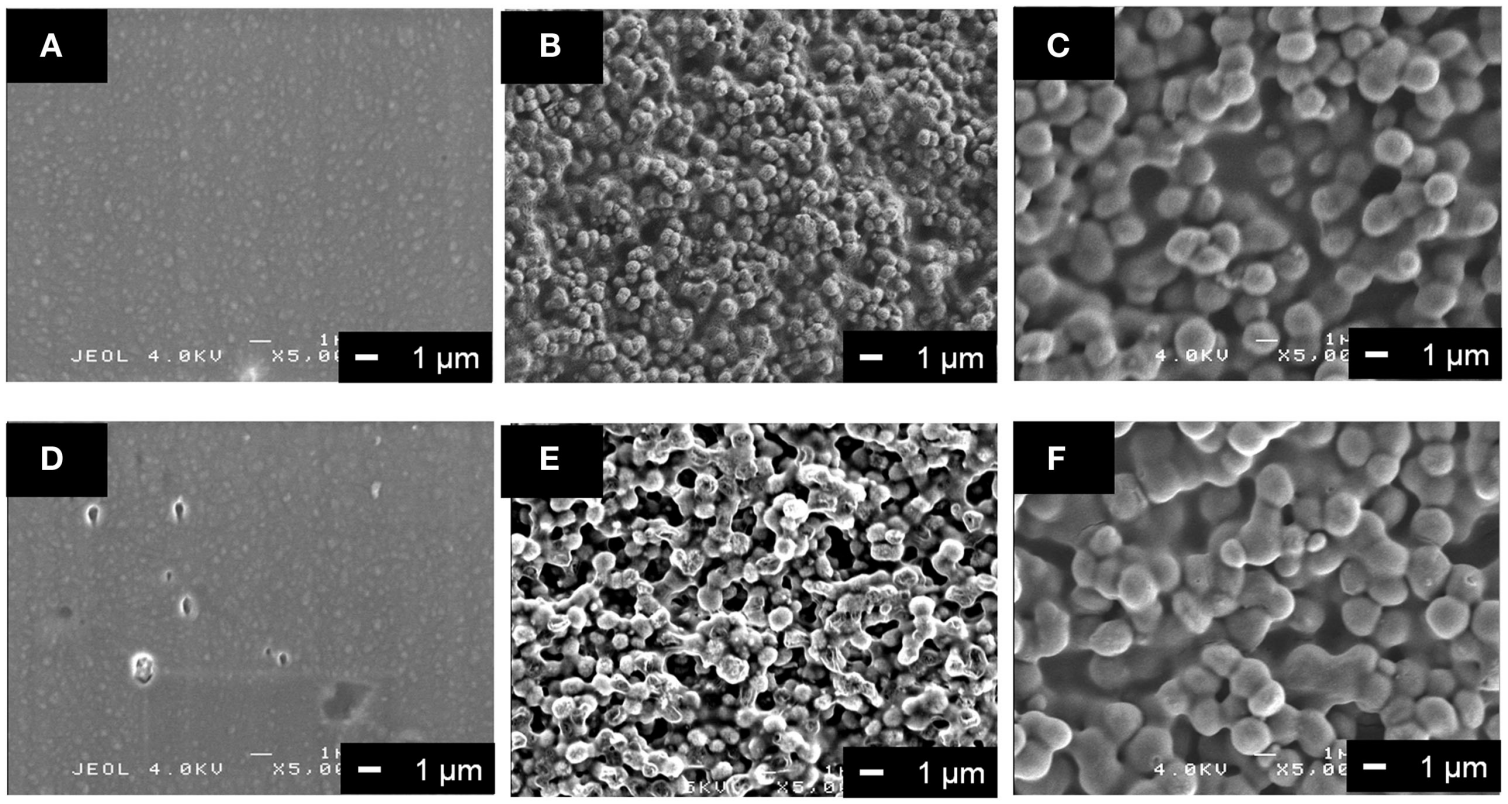
**SEM images of (A,D) PLLA coating, (B,E) PLLA/V coating, and (C,F) PLLA/SiV coating (A–C) before and (D–F) after soaking in α-MEM at 37°C for 7 days**. Reprinted with permission from Yamada et al. (2013).

The surface morphology of the coatings on the metallic Mg was different among the PLLA/SiV, PLLA/V, and pure PLLA (Figures 5A–C), since the diameters of the powders are different; it is ~1.5 μm for SiV and 0.5 μm for V. The thickness also varied among them; it was 5.3 ± 0.4, 3.0 ± 0.1, and 1.8 ± 0.2 μm for PLLA/SiV, PLLA/V, and pure PLLA. Roughnesses of the coatings were 0.40 ± 0.00, 0.19 ± 0.01, and 0.08 ± 0.01 μm for the PLLA/SiV, PLLA/V, and pure PLLA, respectively. This might be due to the difference in the viscosity of the composite or pure PLLA solution. The layer prepared using a spin-coating method depends on the concentration and viscosity of polymer solutions (Schubert and Dunkel, 2003).

Tensile bonding strength tests for the three types of coating demonstrated that the PLLA/SiV coating possesses the highest strength; the rank order of the strength was the PLLA/SiV > PLLA/V > pure PLLA. The difference in the bonding strength would be due to the changes in the molecular weight of PLLA in the coatings by adding the powders. The bonding mechanism was reported to be influenced by molecular weight of polymer in coatings (Xu and Yamamoto, 2012). More free ends of the polymer chains are in the polymers with a lower molecular weight in comparison with those with a higher molecular weight. In the case of the PLLA composite coatings, a larger number of free carboxyl groups for electrostatic intermolecular interaction between polymer chain and the metallic Mg surface is supposed to be contained in PLLA/SiV, based on the results of the tensile bonding tests. The molecular weights are, however, 82 kDa for PLLA/SiV, 46 kDa for PLLA/V, and 90 kDa for pure PLLA. The varied coating thicknesses of the coatings between them might contribute to the differences in the bonding strength.

**Figure 6:**
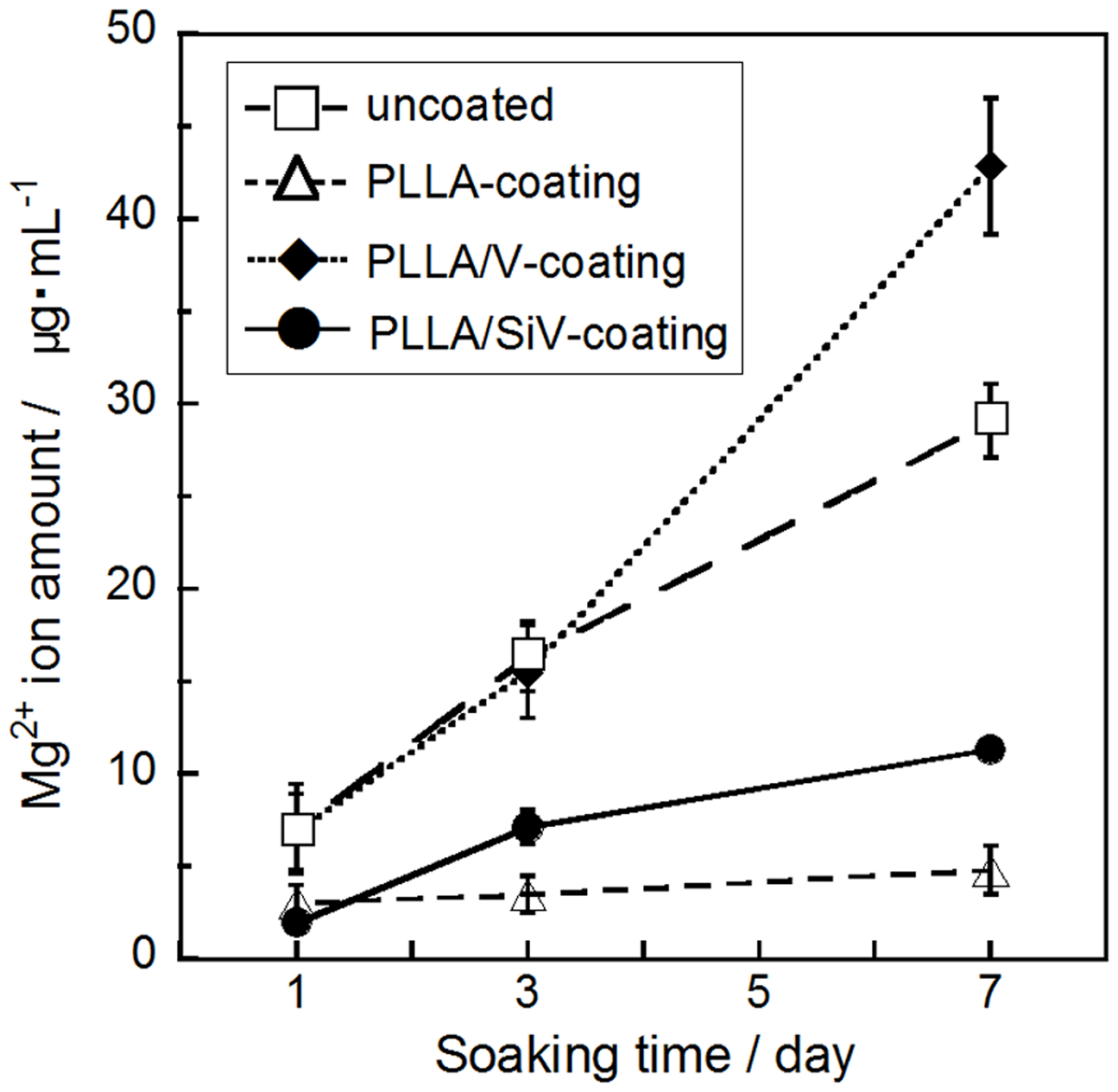
**Mg^2+^ ion concentrations dissolved from uncoated, PLLA coating, PLLA/V coating, and PLLA/SiV coating**. Reprinted with permission from Yamada et al. (2013).

All the coated samples release a trace amount of Mg^2+^ ions in α-MEM, while no detachment of the coating layer from the metallic Mg substrates was happened for them. The amount of the ions significantly decreases by the coatings compared with the uncoated (pure) metallic Mg, except the PLLA/V coating (Figure 6). Up to 30 μg/mL of the ions were released from the uncoated Mg for 7 days of culturing. On the other hand, the PLLA/SiV and PLLA-coated samples released only 11 and 5 μg/mL of the ions, respectively. The PLLA/V-coated sample possessed a completely different releasing behavior from those of the other two samples; the value of the released ions was the same level of the uncoated Mg at day 3 (16 μg/mL) and then reached about 1.4 times as large as that at day 7 (43 μg/mL). This might be because a large amount of pores formed on the surface of the PLLA/V coating, while no pore or tiny one were done on the surfaces of the other two samples (Figures 5D–F). The pores on the PLLA/V coating were generated through the detachment of V powders and the degradation of the PLLA matrix. The Ca^2+^ ions released from the PLLA/V coating might accelerate the corrosion of the metallic Mg, resulting in the enhanced release of Mg^2+^ ion. Thus, the chemical component of the filler in coatings is important for achievement of suppressing the corrosion of the metallic Mg and the rapid release of Mg^2+^ ions from the substrates.

### Cytocompatibility

**Figure 7:**
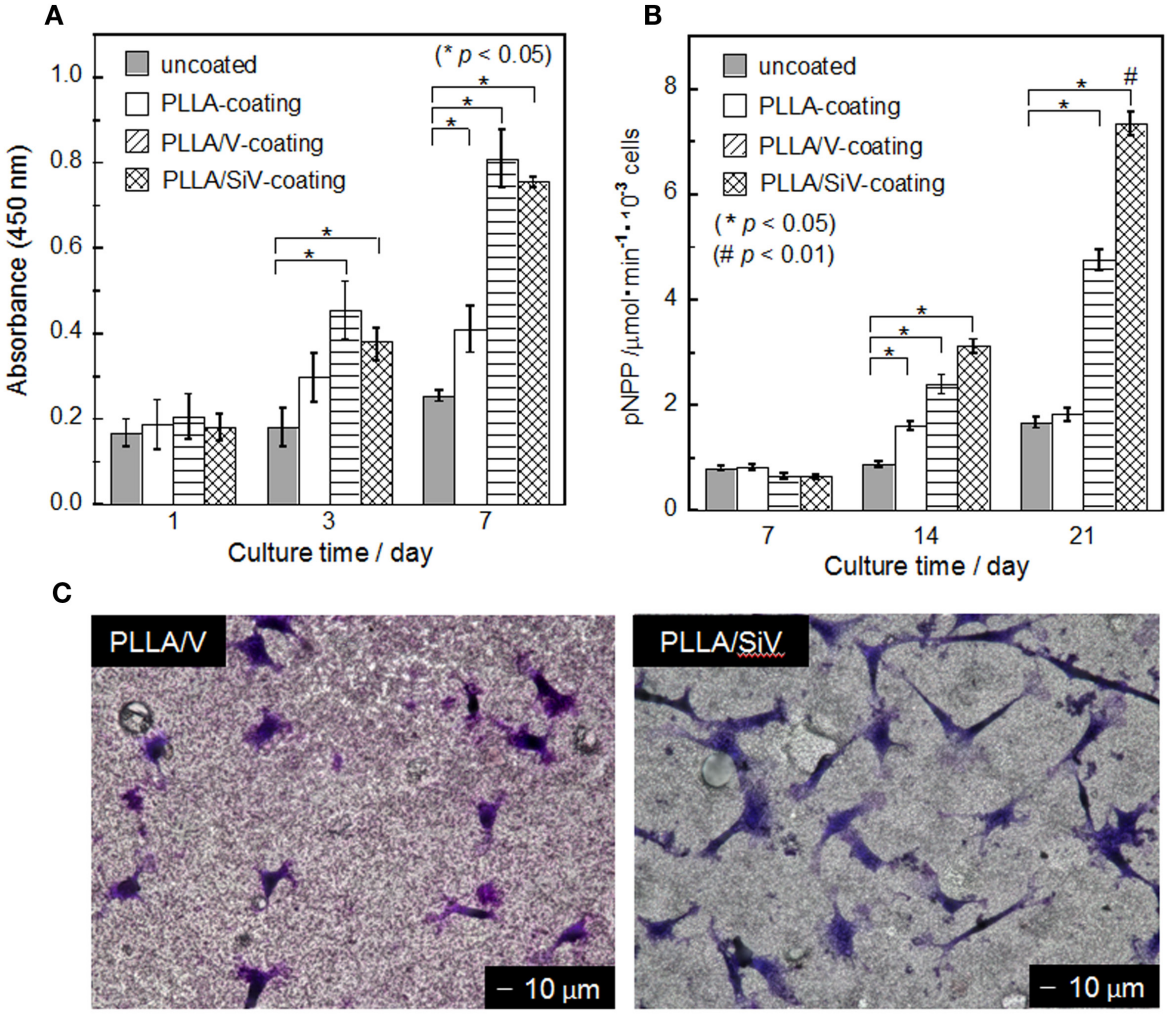
**(A)** Cell number (mean ± SEM; *p < 0.05 as compared to uncoated by *t*-test), **(B)** ALP activity (mean ± SEM; *p < 0.05 as compared to uncoated by *t*-test, ^#^*p* < 0.05 as compared to other three samples by Tukey’s multiple comparison test), and **(C)** morphology of MC3T3-E1 cells cultured on samples. **(C)** After 3 days of culturing. Reprinted with permission from Yamada et al. (2013).

The proliferation of MC3T3-E1 cells on the three types of coated samples and the uncoated one was evaluated by counting live cells after 1, 3, and 7 days of culturing (Figure 7A). Results represent the mean values of the experiments in triplicate. Statistical analysis was performed using Student’s *t*-test and single-factor ANOVA (SPSS 21 software; IBM, USA) followed by Tukey’s multiple comparison test. Values of *p* < 0.05 were considered to be significant. Although almost no proliferation ability was found for the cells cultured on the metallic Mg, the cells proliferated on the coated samples, especially the proliferation on the PLLA/SiV- and PLLA/V-coated samples was excellent. The uncoated sample should degrade rapidly and generate extremely high alkali condition surrounding its surface after seeding the cells, resulting in the poor cell activity. The polymer coatings suppress such harmful influence on the seeded cells by the metallic Mg, which improves the cell activity. The surface morphology and roughness of the coatings might relate to the cell proliferation ability. However, although the PLLA/SiV coating possess much rougher surface than the PLLA/V one, the proliferation ability of the cells was the similar between the two samples. The crystallinity of polymer was also reported to influence cell proliferation (Park and Cima, 1996; Iafisco et al., 2012). The crystallinities of PLLAs in the PLLA/SiV, PLLA/V, and pure PLLA coatings were 12, 9, and 14%, respectively. Thus, the cells on the samples can be regarded to proliferate independently of the crystallinity of the coatings. The Ca^2+^ and Mg^2+^ ions released from the PLLA/SiV- and PLLA/V-coated samples may influence the cell proliferation, since the two ions have been reported to influence osteoblast functions (Diba et al., 2012).

Although there was no significant difference in the proliferation between the PLLA/SiV and PLLA/V coatings, adhering and spreading of the cells varied between the two. The morphology of the cells cultured on the two samples was observed after staining with a Giemsa’s solution (Figure 7C). The cells exhibit spindle-like shape on the PLLA/SiV coating, while they did circular and a less-spread shape on the PLLA/V one. The aspect ratios of the cells varied between the two samples; the ratio of PLLA/SiV samples was higher than that of the PLLA/V ones. The proliferation is comparable between the two samples; nevertheless, the instability of the PLLA/V coatings as shown in Figure 5E might inhibit the cell spreading.

The osteogenic differentiation of the cells varied on the three types of coated samples. The rank order of the ALP activity was the PLLA/SiV > PLLA/V > pure PLLA ≈ the uncoated Mg substrate after 21 days of culturing (Figure 7B). There are two possible reasons why the differentiation varied among the samples, the shape of adhesive cells, and the ions released from the samples. Cell morphologies influence gene expression (Lavenus et al., 2011). As aforementioned, the cells showed a good spreading on the PLLA/SiV coating in comparison with those on the PLLA/V ones. The good spreading should be good for exhibiting their high performances. On the other hand, the ions, especially silicate ions, are known to accelerate osteogenic cell differentiation (Xynos et al., 2001). In addition, MgSiV is expected to possess buffering action in aqueous solution since it releases carbonate ions as well, while most of silica-based bioactive glasses, such as 45S5-type bioactive glass, increase its surrounding pH. This might be good for cells cultured on the material surfaces. Thus, the PLLA/SiV coating is useful for improving the cytocompatibility of the metallic Mg because of its strong bonding with the Mg surface, the stability in an aqueous solution, and the ability of providing three kinds of ions, Mg^2+^, Ca^2+^, and silicate ions, which enhance osteogenic cell functions.

## BONE-VOID Fillers with Cotton Wool-Like Structure and Ion-Providing Ability

Bone-void filler is one of the most common biomaterials for bone reconstruction. Materials for the bone-void fillers are required to have bioactivity and porous structure for achieving excellent cell integration and rapid bone regeneration in body. Electrospun fibremats have been widely investigated for the use in bone tissue engineering because of their flexibility and high interconnected porosity (Li et al., 2002; Sill and von Recum, 2008). A conventional electrospinning system consists of a syringe pomp, a power supply, and a metallic plate (collector). A polymer-based solution is put in a syringe set in the syringe pomp and then electrically charged with the power supply. The electrically charged solution is sprayed onto the earthed collector. Electrospun fibers tightly overlap one another on the collector, resulting in the fabrication of fibremats. However, fabricating thick fibremats, e.g., several millimeter in thickness, had been regarded to be difficult with the conventional electrospinning system, because electrospun fibers hardly sprayed onto a collector when thickness of fibremats formed reaches several hundred micrometer (Pham et al., 2006). Pore sizes of electrospun fibremats are not enough big to induce tissue ingrowth. PLLA/SiV composites having a cotton wool-like structure have been developed with two-types of our original electrospinning systems (Kasuga et al., 2012; Obata et al., 2013). The obtained samples were evaluated in their mechanical properties, ion-releasing ability, and cell compatibility.

### Preparation

Two different systems for electrospinnig were used to fabricate a cotton wool-like structure. One is the system having a vessel (100 mm in diameter) filled with ethanol as a collector (Kasuga et al., 2012). Electrospun fibers are collected in the ethanol, which avoids adhesion between the fibers. In addition, electrical charges on electrospun fibers are expected to be neutralized instantly after entering the ethanol. The electrospun fibers contain large gaps between them, resulting in the formation of 3D structure. Another one is the system having a metallic plate collector (like a conventional system) and a fan which blows air against electrospun fibers (Obata et al., 2013). The air can immediately evaporate the solvent in the electrospun fibers (chloroform) in between a tip of syringe and the collector and prevent the fiber sticking to each other.

### Structure, Ions-Releasing Ability, and Mechanical Properties

**Figure 8:**
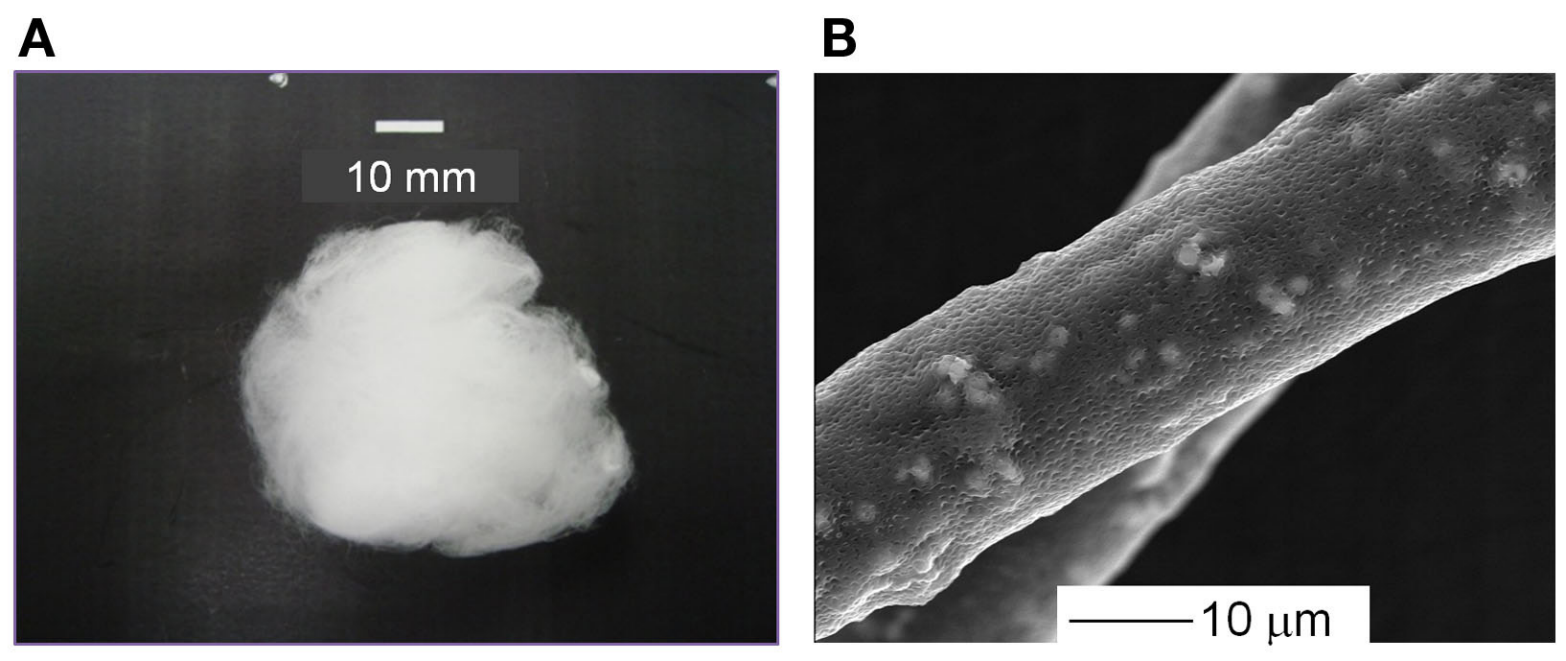
**(A)** Appearance and **(B)** SEM images of cotton wool-like structured PLLA/SiV composites. Reprinted with permission from Obata et al. (2013).

The PLLA composite containing 10, 20, or 30 wt% of SiV with a cotton wool-like structure has been successfully fabricated. The fibers are 10–20 μm in diameter and have pores with ~1 μm in diameter on their surfaces. The SiV powders disperse inside of the fibers and some of them are observed on the fiber surfaces (Figure 8). The pores might be formed due to the evaporation of chloroform in the fibers during the electrospinning (Huang et al., 2003; Kim et al., 2005). The pores are expected to play a role in the achievement of ion releasing from the fibers in an aqueous solution. An aqueous solution must penetrate inside the fibers through the pores, and the SiV powders can be exposed to the solution even at central parts of the fibers. In fact, Ca^2+^ and silicate ions gradually release from the fibers and their amounts depend on the contents of SiV in the composite fibers. Thus, the amounts of the ions released are controllable by changing the content of SiV. When the samples are used as bone-void fillers, they would be tightly packed into irregularly shaped bone defects. Mechanical elasticities of the prepared samples are almost the same as that of the pure PLLA sample. That is, they are able to be packed into such defects without collapse. In addition, handling of the samples during operation must be improved.

### Cell Compatibility

The cotton wool-like structured samples are required to have the ability of cell penetration to achieve rapid bone regeneration inside of them in body. Although the pore size of the samples can be easily varied by mechanically pressing, when their porosity is set to 90 and 96%, the pore sizes in the samples are enough to induce the cell penetration. Results of culture tests using MC3T3-E1 cells for the samples with 90 and 96% in porosity demonstrated that cells successfully migrate into the cotton wool-like structures and proliferate inside of them. In addition, the live cell numbers in the cotton wool-like structures were significantly higher than those on the fibremats. This implies that the cotton wool-like structure can provide a large space allowing the cells to adhere and proliferate.

## Summary

Since inorganic ions were found to stimulate osteogenic cells to proliferate, differentiate, and mineralize, several types of biomaterials releasing such ions have been developed. In this review, we focused on materials releasing three types of ions, Ca^2+^, Mg^2+^, and silicate ions, and their cytocompatibility with osteoblast-like cells. The materials possessed the controlled release of the ions in the culture media. Providing several types of the ions simultaneously was important for achieving enhanced cell functions. Especially, the materials releasing all the three types of ions exhibited higher properties than the others in the results of cell culture tests. Combinatorial effects of inorganic ions provided on cells might exist and are expected to be minutely clarified in the future.

## Conflict of Interest Statement

The authors declare that the research was conducted in the absence of any commercial or financial relationships that could be construed as a potential conflict of interest.

## References

[B1] Bischoff J. L. (1968). Kinetics of calcite nucleation: magnesium ion inhibition and ionic strength catalysis. J. Geophys. Res. 73, 3315–3322. 10.1029/JB073i010p03315

[B2] Böttcher M. E. Gehlken P.-L. Steele D. F. (1997). Characterization of inorganic and biogenic magnesium calcites by Fourier transform infrared spectroscopy. Solid State Ionics. 101–103, 1379–1385. 10.1016/S0167-2738(97)00235-X

[B3] Diba M. Tapia F. Boccaccini A. R. Strobel L. A. (2012). Magnesium-containing bioactive glasses for biomedical applications. Int. J. Appl. Glass Sci. 3, 221–253. 10.1111/j.2041-1294.2012.00095.x

[B4] Falini G. Fermani S. Tosi G. Dinelli E. (2009). Calcium carbonate morphology and structure in the presence of seawater ions and humic acids. Cryst. Growth Des. 9, 2065–2072. 10.1021/cg8002959

[B5] Falini G. Gazzano M. Ripamonti A. (1996). Magnesium calcite crystallizatin from water-alcohol mixtures. Chem. Commun. 9, 1037–1038. 10.1039/cc9960001037

[B6] Fujikura K. Lin S. Nakamura J. Obata A. Kasuga T. (2013). Preparation of electrospun fiber mats using siloxane-containing vaterite and biodegradable polymer hybrids for bone regeneration. J. Biomed. Mater. Res. B Appl. Biomater. 101, 1350–1358. 10.1002/jbm.b.32952 23687079

[B7] Hoppe A. Güldal N. S. Boccaccini A. R. (2011). A review of the biological response to ionic dissolution products from bioactive glasses and glass-ceramics. Biomaterials 32, 2757–2774. 10.1016/j.biomaterials.2011.01.004 21292319

[B8] Huang Z.-M. Zhang Y.-Z. Kotaki M. Ramakrishna S. (2003). A review on polymer nanofibers by electrospinning and their applications in nanocomposites. Compos. Sci. Technol. 63, 2223–2253. 10.1016/S0266-3538(03)00178-7

[B9] Iafisco M. Palazzo B. Ito T. Otsuka M. Senna M. Delgado-Lopez J. M. (2012). Preparation of core–shell poly(l-lactic) acid-nanocrystalline apatite hollow microspheres for bone repairing applications. J. Mater. Sci. Mater. Med. 23, 2659–2669. 10.1007/s10856-012-4732-1 22864504

[B10] Jarcho M. (1981). Calcium phosphate ceramics as hard tissue prosthetics. Clin. Orthop. Relat. Res. 157, 259–278. 7018783

[B11] Kasuga T. Obata A. Maeda H. Ota Y. Yao X. Oribe K. (2012). Siloxane-poly(lactic acid)-vaterite composites with 3D cotton-like structure. J. Mater. Sci. Mater. Med. 23, 2349–2357. 10.1007/s10856-012-4607-5 22415363

[B12] Kim G.-T. Lee J.-S. Shin J.-H. Ahn Y.-C. Hwang Y.-J. Shin H.-S. (2005). Investigation of pore formation for polystyrene electrospun fiber: effect of relative humidity. Korean J. Chem. Eng. 22, 783–788. 10.1007/BF02705799

[B13] Kitamura M. (2001). Crystallization and transformation mechanism of calcium carbonate polymorphs and the effect of magnesium ion. J. Colloid Interface Sci. 236, 318–327. 10.1006/jcis.2000.7398 11401379

[B14] Kitano Y. (1962). The behavior of various inorganic ions in the separation of calcium carbonate from a bicarbonate solution. Bull. Chem. Soc. Jpn. 35, 1973–1980. 10.1246/bcsj.35.1973

[B15] Lavenus S. Berreur M. Trichet V. Pilet P. Louarn G. Layrolle P. (2011). Adhesion and osteogenic differentiation of human mesenchymal stem cells on titanium nanopores. Eur. Cell. Mater. 22, 84–96. 21870339 10.22203/ecm.v022a07

[B16] LeGeros R. Z. (2002). Properties of osteoconductive biomaterials: calcium phosphates. Clin. Orthop. Relat. Res. 395, 81–98. 10.1097/00003086-200202000-00009 11937868

[B17] Li W. J. Laurencin C. T. Caterson E. J. Tuan R. S. Ko F. K. (2002). Electrospun nanofibrous structure: a novel scaffold for tissue engineering. J. Biomed. Mater. Res. 60, 613–621. 10.1002/jbm.10167 11948520

[B18] Maeno S. Niki Y. Matsumoto H. Morioka H. Yatabe T. Funayama A. (2005). The effect of calcium ion concentration on osteoblast viability, proliferation and differentiation in monolayer and 3D culture. Biomaterials 26, 4847–4855. 10.1016/j.biomaterials.2005.01.006 15763264

[B19] Maier J. A. Bernardini D. Rayssiguier Y. Mazur A. (2004). High concentrations of magnesium modulate vascular endothelial cell behaviour in vitro. Biochim. Biophys. Acta 1689, 6–12. 10.1016/j.bbadis.2004.02.004 15158908

[B20] Morse J. W. Wang Q. Tsio M. Y. (1997). Influences of temperature and Mg:Ca ratio on CaCO3 precipitates from seawater. Geology 25, 85–87. 10.1130/0091-7613(1997)025<0085:IOTAMC>2.3.CO;2

[B21] Nakamura J. Poologasundarampillai G. Jones J. R. Kasuga T. (2013). Tracking the formation of vaterite particles containing aminopropyl-functionalized silsesquioxane and their structure for bone regenerative medicine. J. Mater. Chem. B 1, 4446–4454. 10.1039/c3tb20589d 32261117

[B22] Obata A. Hotta T. Wakita T. Ota Y. Kasuga T. (2010). Electrospun microfiber meshes of silicon-doped vaterite/poly(lactic acid) hybrid for guided bone regeneration. Acta Biomater. 6, 1248–1257. 10.1016/j.actbio.2009.11.013 19913116

[B23] Obata A. Ozasa H. Kasuga T. Jones J. R. (2013). Cotton wool-like poly(lactic acid)/vaterite composite scaffolds releasing soluble silica for bone tissue engineering. J. Mater. Sci. Mater. Med. 24, 1649–1658. 10.1007/s10856-013-4930-5 23606191

[B24] Obata A. Tokuda S. Kasuga T. (2009). Enhanced in vitro cell activity on silicon-doped vaterite/poly(lactic acid) composites. Acta Biomater. 5, 57–62. 10.1016/j.actbio.2008.08.004 18786869

[B25] Park A. Cima L. G. (1996). In vitro cell response to differences in poly-L-lactide crystallinity. J. Biomed. Mater. Res. 31, 117–130. 10.1002/jbm.1996.820310102 8731156

[B26] Pham Q. P. Sharma U. Mikos A. G. (2006). Electrospun poly (ε-caprolactone) microfiber and multilayer nanofiber/microfiber scaffolds: characterization of scaffolds and measurement of cellular infiltration. Biomacromolecules 7, 2796–2805. 10.1021/bm060680j 17025355

[B27] Saboori A. Rabiee M. Moztarzadeh F. Sheikhi M. Tahriri M. Karimi M. (2009). Synthesis, characterization and in vitro bioactivity of sol-gel-derived SiO_2_-CaO-P_2_O_5_-MgO bioglass. Mater. Sci. Eng. C 29, 335–340. 10.1016/j.msec.2008.07.004

[B28] Sawada K. Ogino T. Suzuki T. (1990). The distribution coefficients of Mg^2+^ ion between CaCO_3_ polymorphs and solution and the effects on the formation and transformation of CaCO_3_ in water. J. Cryst. Growth 106, 393–399. 10.1016/0022-0248(90)90084-X

[B29] Schubert D. Dunkel T. (2003). Spin coating from a molecular point of view: its concentration regimes, influence of molar mass and distribution. Mater. Res. Innovat. 7, 314–321. 10.1007/s10019-003-0270-2

[B30] Shen Y. Liu W. Wen C. Pan H. Wang T. Darvell B. W. (2012). Bone regeneration: importance of local pH-strontium-doped borosilicate scaffold. J. Mater. Chem. 22, 8662–8670. 10.1039/c2jm16141a

[B31] Sill T. J. von Recum H. A. (2008). Electrospinning: applications in drug delivery and tissue engineering. Biomaterials 29, 1989–2006. 10.1016/j.biomaterials.2008.01.011 18281090

[B32] Staiger M. P. Pietak A. M. Huadmai J. Dias G. (2006). Magnesium and its alloys as orthopedic biomaterials: a review. Biomaterials 27, 1728–1734. 10.1016/j.biomaterials.2005.10.003 16246414

[B33] Wakita T. Obata A. Poologasundarampillai G. Jones J. R. Kasuga T. (2010). Preparation of electrospun siloxane-poly(lactic acid)-vaterite hybrid fibrous membranes for guided bone regeneration. Compos. Sci. Technol. 70, 1889–1893. 10.1016/j.compscitech.2010.05.014

[B34] Wang J. Becker U. (2009). Structure and carbonate orientation of vaterite (CaCO3). Am. Mineral. 94, 380–386. 10.2138/am.2009.2939

[B35] Winter M. Griss P. de Groot K. Tagai H. Heimke G. von Dijk H. J. (1981). Comparative histocompatibility testing of seven calcium phosphate ceramics. Biomaterials 2, 159–IN151. 10.1016/0142-9612(81)90043-0 6268208

[B36] Witte F. (2010). The history of biodegradable magnesium implants: a review. Acta Biomater. 6, 1680–1692. 10.1016/j.actbio.2010.02.028 20172057

[B37] Witte F. Hort N. Vogt C. Cohen S. Ulrich Kainer K. Willumeit R. (2008). Degradable biomaterials based on magnesium corrosion. Curr. Opin. Solid State Mater. Sci. 12, 63–72. 10.1016/j.cossms.2009.04.001

[B38] Witte F. Kaese V. Haferkamp H. Switzer E. Meyer-Lindenberg A. Wirth C. J. (2005). In vivo corrosion of four magnesium alloys and the associated bone response. Biomaterials 26, 3557–3563. 10.1016/j.biomaterials.2004.09.049 15621246

[B39] Wong H. M. Yeung K. W. Lam K. O. Tam V. Chu P. K. Luk K. D. (2010). A biodegradable polymer-based coating to control the performance of magnesium alloy orthopaedic implants. Biomaterials 31, 2084–2096. 10.1016/j.biomaterials.2009.11.111 20031201

[B40] Xu L. Yamamoto A. (2012). Characteristics and cytocompatibility of biodegradable polymer film on magnesium by spin coating. Colloids Surf. B Biointerfaces 93, 67–74. 10.1016/j.colsurfb.2011.12.009 22225942

[B41] Xynos I. D. Edgar A. J. Buttery L. D. Hench L. L. Polak J. M. (2000a). Ionic products of bioactive glass dissolution increase proliferation of human osteoblasts and induce insulin-like growth factor II mRNA expression and protein synthesis. Biochem. Biophys. Res. Commun. 276, 461–465. 10.1006/bbrc.2000.3503 11027497

[B42] Xynos I. D. Hukkanen M. V. Batten J. J. Buttery L. D. Hench L. L. Polak J. M. (2000b). Bioglass ^®^45S5 stimulates osteoblast turnover and enhances bone formation in vitro: implications and applications for bone tissue engineering. Calcif. Tissue Int. 67, 321–329. 10.1007/s002230001134 11000347

[B43] Xynos I. D. Edgar A. J. Buttery L. D. Hench L. L. Polak J. M. (2001). Gene-expression profiling of human osteoblasts following treatment with the ionic products of Bioglass^®^ 45S5 dissolution. J. Biomed. Mater. Res. 55, 151–157. 10.1002/1097-4636(200105)55:2<151::AID-JBM1001>3.0.CO;2-D 11255166

[B44] Yamada S. Maeda H. Obata A. Lohbauer U. Yamamoto A. Kasuga T. (2013). Cytocompatibility of siloxane-containing vaterite/poly(_L_-lactic acid) composite coatings on metallic magnesium. Materials 6, 5857 10.3390/ma6125857 PMC5452738 28788425

[B45] Yamada S. Ota Y. Nakamura J. Sakka Y. Kasuga T. (2014a). Preparation of siloxane-containing vaterite doped with magnesium. J. Ceram. Soc. Jpn. 122, 1010–1015. 10.2109/jcersj2.122.1010

[B46] Yamada S. Yamamoto A. Kasuga T. (2014b). Poly(_L_-lactic acid)/vaterite composite coatings on metallic magnesium. J. Mater. Sci. Mater. Med. 25, 2639–2647. 10.1007/s10856-014-5302-5 25096227

[B47] Zhang Z. Xie Y. Xu X. Pan H. Tang R. (2012). Transformation of amorphous calcium carbonate into aragonite. J. Cryst. Growth 343, 62–67. 10.1016/j.jcrysgro.2012.01.025

[B48] Zreiqat H. Howlett C. R. Zannettino A. Evans P. Schulze-Tanzil G. Knabe C. (2002). Mechanisms of magnesium-stimulated adhesion of osteoblastic cells to commonly used orthopaedic implants. J. Biomed. Mater. Res. 62, 175–184. 10.1002/jbm.10270 12209937

